# Differences in the timing and magnitude of *Pkd1* gene deletion determine the severity of polycystic kidney disease in an orthologous mouse model of ADPKD

**DOI:** 10.14814/phy2.12846

**Published:** 2016-06-29

**Authors:** Kelly A. Rogers, Sarah E. Moreno, Laurie A. Smith, Hervé Husson, Nikolay O. Bukanov, Steven R. Ledbetter, Yeva Budman, Yuefeng Lu, Bing Wang, Oxana Ibraghimov‐Beskrovnaya, Thomas A. Natoli

**Affiliations:** ^1^ Department of Rare Renal Disease ResearchSanofi‐Genzyme R&D CenterFraminghamMassachusetts; ^2^ Department of Analytical Research and DevelopmentSanofi CorporationWalthamMassachusetts; ^3^ Department of Biostatistics and ProgrammingSanofi‐Genzyme R&D CenterFraminghamMassachusetts; ^4^ Present address: BioRepair ConsultantsWestboroughMassachusetts

**Keywords:** Cell cycle progression, cyst growth, glycosphingolipid, mouse model, polycystic kidney disease

## Abstract

Development of a disease‐modifying therapy to treat autosomal dominant polycystic kidney disease (ADPKD) requires well‐characterized preclinical models that accurately reflect the pathology and biochemical changes associated with the disease. Using a *Pkd1* conditional knockout mouse, we demonstrate that subtly altering the timing and extent of *Pkd1* deletion can have a significant impact on the origin and severity of kidney cyst formation. *Pkd1* deletion on postnatal day 1 or 2 results in cysts arising from both the cortical and medullary regions, whereas deletion on postnatal days 3–8 results in primarily medullary cyst formation. Altering the extent of *Pkd1* deletion by modulating the tamoxifen dose produces dose‐dependent changes in the severity, but not origin, of cystogenesis. Limited *Pkd1* deletion produces progressive kidney cystogenesis, accompanied by interstitial fibrosis and loss of kidney function. Cyst growth occurs in two phases: an early, rapid growth phase, followed by a later, slow growth period. Analysis of biochemical pathway changes in cystic kidneys reveals dysregulation of the cell cycle, increased proliferation and apoptosis, activation of Mek‐Erk, Akt‐mTOR, and Wnt‐*β*‐catenin signaling pathways, and altered glycosphingolipid metabolism that resemble the biochemical changes occurring in human ADPKD kidneys. These pathways are normally active in neonatal mouse kidneys until repressed around 3 weeks of age; however, they remain active following *Pkd1* deletion. Together, this work describes the key parameters to accurately model the pathological and biochemical changes associated with ADPKD in a conditional mouse model.

## Introduction

Autosomal dominant polycystic kidney disease (ADPKD) is characterized by the bilateral development of fluid‐filled kidney cysts that progressively destroy the renal tubular architecture (Harris and Torres 2009). ADPKD is caused by mutations in either the *PKD1* (85%) or *PKD2* (15%) genes (Consortium. TIPKD, 1995; Hughes et al. 1995; Mochizuki et al. 1996; Schneider et al. 1996); disease severity varies depending on environmental factors or genetic differences (Harris and Rossetti 2010). An estimated 50% of patients develop end‐stage renal disease by their sixth decade due to progressive cyst expansion and the development of interstitial fibrosis in both kidneys (Grantham et al. 2011; Takiar and Caplan 2011). Extrarenal manifestations include liver and pancreatic cysts, as well as cardiovascular abnormalities (Pirson 2010). Advances in our understanding of cellular pathways that drive cyst growth have provided a number of potential therapeutic targets. These pathways include cilia–cell cycle regulation, apoptosis, cAMP, Ca^2+^ signaling, Src, mitogen‐activated kinase/extracellular‐regulated kinase (MAPK/ERK) signaling, and Akt/mTOR signaling cascades (Nadasdy et al. 1995; Nagao et al. 2003; Yamaguchi et al. 2003; Bukanov et al. 2006; Omori et al. 2006; Smith et al. 2006; Ibrahim 2007; Park et al. 2007; Ibraghimov‐Beskrovnaya and Natoli 2011). Importantly, targeting key elements of these pathways slows disease progression in preclinical models of PKD, demonstrating the importance of these pathways to cyst growth and providing disease‐modifying therapeutic options for individuals afflicted with ADPKD (Tao 2005; Tao et al. 2005; Bukanov et al. 2006; Omori et al. 2006; Wahl et al. 2006; Wu et al. 2007; Gattone et al. 2009; Zafar et al. 2009; Shillingford et al. 2010). Clinical trials targeting specific proteins within these pathways are currently underway or have recently been completed (Torres et al. 2012; Perico et al. 2010; Serra et al. 2010; Walz et al. 2010).

Preclinical testing of therapeutics requires relevant, well‐characterized animal models. Currently, there is no widely accepted single model of ADPKD for therapeutic testing, so most agents are tested in multiple preclinical models (Menezes and Germino 2013). Since human ADPKD involves a slow progression of cyst growth over decades, a genetically orthologous animal model that develops slowly progressive kidney cystogenesis in adulthood would have the most relevant pathology. Toward this end, several mouse models of human ADPKD have been developed. Mice heterozygous for germline *Pkd1* or *Pkd2* mutations are genetically similar to humans with ADPKD, but either lack a cystic phenotype or develop extremely mild cystogenesis after a long period (Lu et al. 1999; Wu et al. 2002). Mice homozygous for germline *Pkd1* or *Pkd2* mutations develop kidney and pancreatic cysts in utero; however, these mutations are embryonic lethal, preventing studies in adult mice (Lu et al. 1997; Wu et al. 1998; Boulter et al. 2001). To circumvent the problem of embryonic lethality, several models have been developed that inactivate *Pkd1* or *Pkd2* function tissue specifically, randomly in a small number of cells, or in response to chemical induction (Piontek 2004; Jiang et al. 2006; Lantinga‐van Leeuwen et al. 2007; Piontek et al. 2007; Starremans et al. 2008; Takakura et al. 2008; Shillingford et al. 2010). Kidney‐specific inactivation of *Pkd1* results in an aggressive cystic phenotype, leading to death within the first few weeks of life (Lantinga‐van Leeuwen et al. 2007). Random inactivation of *Pkd1* or *Pkd2* in a subset of cells, such as in the *Pkd2* ^*ws25/‐*^ and *Pkd1* ^*cond/cond*^ *:Nestin* ^*cre*^ mouse lines, causes cystogenesis in adult kidneys and are pathologically similar to ADPKD (Wu et al. 1998; Shillingford et al. 2010). However, the unpredictable nature of the deletion in these models can result in heterogeneity and a slow rate of progression, making them less attractive for therapeutic testing. *Pkd1* and *Pkd2* conditional knockout (cKO) mouse lines, coupled with inducible Cre recombinases, offer an alternative model (Lantinga‐van Leeuwen et al. 2007; Piontek et al. 2007; Takakura et al. 2008; Kim et al. 2009; Leonhard et al. 2015). Neonatal deletion of *Pkd1* (within the first 12 days after birth) results in aggressive kidney cystogenesis within a few weeks of *Pkd1* loss; however, when *Pkd1* is deleted after this period, mild cystogenesis develops after 6–9 months (Lantinga‐van Leeuwen et al. 2007; Piontek et al. 2007; Takakura et al. 2008; Leonhard et al. 2015). While these models are all genetically relevant to ADPKD, the pathology is either very aggressive (embryonic or neonatal), or very slowly progressive, often with a high level of heterogeneity.

In order to develop a *Pkd1*‐linked model of ADPKD that recapitulates the key pathological and biochemical features of the disease within a timeframe suitable for therapeutic testing, we tested the effects of altering the timing and dose of tamoxifen in an inducible *Pkd1* cKO mouse model. Here, we demonstrate that subtle changes in the timing of *Pkd1* deletion can modify the origin of the resulting cysts, while changes in the dose of tamoxifen influence the extent of cystogenesis. We describe conditions that result in the progressive development of both cortical and medullary cysts, interstitial fibrosis, and a slow renal functional decline in adult mice, similar to that occurring in human ADPKD. Renal cyst growth occurs rapidly between 18 and 26 days of age, and then slows at later times. Biochemical pathways that drive cystogenesis, including cell cycle progression, apoptosis, mitogenic signaling, Akt‐mTOR activity, and Wnt signaling activity, are changed similarly in the *Pkd1* cKO and human ADPKD kidneys. Moreover, we demonstrate that these pathways are active during the neonatal period, and loss of *Pkd1* prevents the developmental shutdown of these pathways that occurs during normal neonatal kidney maturation. We further demonstrate that increases in glycosphingolipid accumulation, which reflect the increases observed in human ADPKD, begin soon after the onset of cystogenesis. Thus, we have developed and characterized an adult, *Pkd1*‐linked model of cystogenesis that should be useful for the disease analysis and therapeutic testing.

## Materials and Methods

### Animal handling

Generation of the *Pkd1* conditional knockout (*Pkd1* ^*tm1Gztn*^, with lox sites flanking exons 21–23), the *Pkd1* germline null (*Pkd1* ^*tm1Gzbd*^, with a duplication of exons 15–23 leading to a frameshift and truncation), and the Cre deleter (*CreER* ^*T2*^) mouse strains used in these studies have been previously described (Seibler et al. 2003; Natoli et al. 2008, 2010). To induce Cre recombinase activity, we delivered tamoxifen in sunflower oil (both from Sigma‐Aldrich, St. Louis, MO), at the doses and times indicated in the text, to nursing females by intraperitoneal injection. The nursing females subsequently transmitted tamoxifen‐containing milk to their pups. Mouse colonies were maintained at Charles River Laboratories (Wilmington, MA) or Sanofi‐Genzyme (Framingham, MA) and handled in accordance with protocols approved by the respective Institutional Animal Care and Use Committees.

### Genotyping

DNA was isolated from tail samples as described elsewhere (Laird et al. 1991). All PCR‐based genotyping was performed using Platinum *Taq* DNA polymerase (Invitrogen, Carlsbad, CA). Amplification of the conditional *Pkd1* allele was achieved in the presence of 1.5 mmol/L MgCl_2_ with primers: 5′‐GGGCTAACGCAGCAGTAATC‐3′ and 5′‐CCCTCAGCATGTCATCGAGT‐3′. The cycling parameters for conditional *Pkd1* amplification were as follows: 94°C × 5′, 35 × (94°C × 30″, 57°C × 30″, 72°C × 1′), 72°C × 5′, 4°C hold. PCR detection of the Cre allele in the *CreER* ^*T2*^‐containing DNA samples was performed in the presence of 3.0 mmol/L MgCl_2_ with the following primer sequences: 5′‐CTCTTCCCTCGTGATCTGCAACTCC‐3′; 5′‐CATGTCTTTAATCTACCTCGATGG‐3′; 5′‐ATGCAACGAGTGATGAGGTT‐3′; and 5′‐CTCGACCAGTTTAGTTACCC‐3′. PCR cycling was conducted as follows: 94°C × 2′, 30 × (94°C × 30″, 60°C × 30″, 72°C × 30″), 72°C × 4′, 4°C hold. Agarose gel electrophoresis and visualization of the PCR products was carried out using 2% NuSieve agarose gels in the presence of ethidium bromide. PCR‐based genotyping of the *Pkd1* ^*tm1Gzbd*^ mutation was performed as described previously (Natoli et al. 2008).

### Quantification of deletion efficiency

Kidney DNA was isolated from *Pkd1* ^*tm1Gztn/+*^:Cre+ mice (littermate controls to the cystic animals described herein) exposed to tamoxifen as described above. Littermate control animals were used to avoid the complications due to the duplication of this region in the *Pkd1* ^*tm1Bdgz*^ germline knockout allele. Droplet digital PCR was used to quantify the presence of exon 16 (outside of the floxed sequence) and a portion of intron 23 (contained within the floxed region) in a single reaction (Hindson et al. 2011). Primer sets: exon 16; amplification primers, 5′‐GGTGATCAGACACCGCTCAA‐3′ and 5′‐GACCCACCCCAGGAATAACC‐3′, probe, 5′‐VIC‐ CTGCGTGGCTTCAACACAGGTCAGC‐TAMRA‐3′; intron 23; amplification primers, 5′‐GGTCCTAGGGGTCTGGCTAA‐3′ and 5′‐AGGGCCATTTAAGCAGAGGAC‐3′, probe, 5′‐FAM‐CCTGTGGCCAGAGAGAAGCATGTGTTG‐TAMRA‐3′. A total quantity of 25 *μ*L reactions containing 100 ng genomic DNA, 11.25 pmol of each amplification primer, and 6.25 pmol of each probe oligonucleotide in 1× ddPCR Supermix for probes without dUTP (Bio‐Rad, Hercules, CA) were used for droplet formation (performed in a Bio‐Rad automated droplet generator following manufacturer's instructions). PCR reactions were performed as follows: 95°C × 10′; 40 × [94°C × 30″; 61°C, 1′]; 98°C × 10′; 4°C hold. PCR reactions were quantified on a Bio‐Rad QX200 droplet reader. For each reaction, the ratio of the absolute count of the intron 23 to exon 16 was determined. DNA was isolated from three independent animals for each deletion scheme; each sample was assayed in duplicate, in three independent experiments (six values per sample total). The percent deletion was calculated using the following formula: % deletion = (1 − [mean ratio test sample]/[mean ratio wild‐type DNA])*100*2); multiplying by 2 compensated for the presence of the wild‐type allele in these samples.

### Histological analysis

Samples for histological processing were fixed for 48 hours in 4% paraformaldehyde in phosphate‐buffered saline (PBS) before paraffin embedding using standard procedures. Routine staining of kidney sections with hematoxylin and eosin (H&E) and Masson's trichrome were performed. No gender difference in the extent of cystogenesis was observed; therefore, males and females were pooled for analysis.

### Cyst quantification

Cyst percentage was calculated from H&E‐stained kidney sections using MetaMorph^™^ image analysis software (Molecular Devices, Sunnyvale, CA) as described previously (Smith et al. 2006). To estimate cyst volume, cyst percentage was multiplied by total kidney weight to determine total cyst weight, which was then converted to total cyst volume by assuming 1 g/mL of cyst fluid.

### Ki67 immunohistochemical staining

Paraffin‐embedded tissue sections were deparaffinized through a xylene/graded ethanol series following standard histochemical procedures; antigen retrieval was performed by heat treating in a citrate buffer (Dako North America, Inc., Carpinteria, CA) for 20–30 min using a pressure cooker. Slides were blocked with a peroxidase‐blocking solution (Dako), preincubated with a serum‐free protein blocking agent (Dako), and incubated with an anti‐Ki67 antibody (Abcam, Cambridge, MA) diluted in an antibody diluent reagent (Dako) at 4°C overnight. Primary antibody binding was revealed using an Envision+ HRP system followed by 3,3′‐diaminobenzidine tetrahydrochloride (Dako) following the manufacturer's recommendations, and the slides were counterstained with hematoxylin prior to mounting. For quantification of Ki67 staining, slides from four representative animals per group were digitized using a Chromavision ACIS II scanner, and the total and Ki67‐positive nuclei from cyst‐lining epithelial cells from 40 to 50 cysts per animal were counted manually.

### Blood urea nitrogen measurement

Blood was collected into serum separator tubes (BD, Franklin Lakes, NJ) at killing by cardiac puncture, and serum was isolated after centrifugation. Levels of serum blood urea nitrogen (BUN) were determined by ACE BUN assay on a VetACE^™^ bioanalyzer (Alfa Wassermann, West Caldwell, NJ).

### Immunoblot analysis

Whole kidneys were homogenized using an Omni Beadbeater 24 homogenizer (Omni International, Kennesaw, GA; speed = 5.65, duration 2 × 30 sec) at 4°C in RIPA lysis buffer (Boston BioProducts, Ashland, MA) containing 1 mmol/L DTT, 5 mmol/L EDTA, 2 mmol/L NaF, 1 mmol/L Na_3_VO_4_, 1× Pefablock^™^ (Roche Applied Science, Indianapolis, IN), and 1× complete Protease Inhibitor Cocktail^™^ (Roche Applied Science). Total protein concentration was determined using a BCA assay kit (Pierce, part of Thermo Fisher Scientific, Rockford, IL) following the manufacturer's protocol. Equal amounts of protein (40 *μ*g) were electrophoresed on 4–12% Bis‐Tris SDS‐PAGE gels and transferred to nitrocellulose using the iBlot^™^ dry transfer system (Invitrogen, Carlsbad, CA). Membranes were blocked with 5% nonfat dry milk in 1× TBS/Tween‐20 (Boston BioProducts) and primary antibodies were incubated overnight at 4°C. The following primary antibodies were used: proliferating cell nuclear antigen (PCNA; Dako); Cyclin D1, total Erk1/2, phosphorylated Erk1/2 (Thr202/Tyr204), phosphorylated Akt (Ser473), phosphorylated GSK3*β* (Ser9), phosphorylated S6 ribosomal protein (Ser235/236, human only), total S6 ribosomal protein, total Smad2, total Smad3 (all from Cell Signaling, Danvers, MA); Apaf‐1 (BD Biosciences, San Jose, CA); cMyc (Abcam, Cambridge, MA); Bcl‐xL, total GSK3*β*, total *β*‐catenin (BD Transduction, Lexington, KY); Caspase 2 (Abcam for detection of human antigen; BD Transduction for detection of mouse antigen); Bad, Akt (BD Biosciences); Mek, phosphorylated S6 ribosomal protein (Ser235) (mouse only; EMD Millipore, Billerica, MA); phosphorylated Smad3 (Ser423/425) (Novus Biologicals, Littleton, CO); Smad7 (Santa Cruz Biotechnology, Santa Cruz, CA). Protein loading was controlled using anti‐beta Actin (Abcam). Membranes were washed in 1× TBS/Tween‐20 and incubated for 1 h with secondary antibodies conjugated to horseradish peroxidase (HRP): anti‐mouse IgG‐HRP or anti‐rabbit IgG‐HRP at 1:10,000 dilution (Promega, Madison, WI); anti‐rabbit IgG‐HRP at 1:5000 dilution for binding to anti‐cMyc (Cell Signaling). Blots were visualized using enhanced chemiluminescence (GE Healthcare, Little Chalfont, UK; Thermo Fisher Scientific, Waltham, MA). Human kidney samples were acquired as previously described (Natoli et al. 2010).

### ELISA analysis

Kidneys were homogenized using an Omni Beadbeater 24 homogenizer (Omni International; speed = 5.65, duration 2 × 30 sec) at 4°C in 1.65 mol/L NaCl, 50 mmol/L Tris pH 7.4. Total protein concentration was determined using a BCA assay kit (Pierce) as described by the manufacturer. Total or active PAI1 ELISAs were performed using ELISA kits from Molecular Innovations, Inc (Novi, MI) as described by the manufacturer.

### Glycosphingolipid analysis

Acquisition of human kidney samples and analysis of glycosphingolipid levels was previously described (Natoli et al. 2010). Quantitative analysis of mouse kidney sphingolipids was performed by liquid chromatography and tandem mass spectrometry (LC/MS/MS). Briefly, kidney tissue was homogenized five times its volume of water (w/v) by a bead beater. Glucosylceramide (GlcCer), galactosylceramide, and lactosylceramide (LacCer) were extracted by adding 1 mL of mobile phase A (MPA) and mobile phase B (MPB) (9:1, vol/vol) to 20 *μ*L of the homogenate. MPA consisted of acetonitrile, methanol, acetic acid, and water at a volume ratio of 96/2/2/1, and 5 mmol/L ammonium acetate. MPB consisted of methanol, acetic acid, and water at a volume ratio of 98/1/1, and 5 mmol/L ammonium acetate. Ceramide and GM3 were extracted by adding 1 mL of MPA and MPB (1:1, vol/vol) to 40 *μ*L of the homogenate. PC was coextracted with the sphingolipids and used for normalization. The samples were placed on a VX‐2500 tube vortexer (VWR International, LLC, Radnor, PA) for 3 min at a speed of 10 and then centrifuged for 4 min at 10,000 g. The resultant supernatant was transferred into HPLC vials for analysis. For the analysis of PC, the supernatant was diluted in MPA by 50–100‐fold to bring the signals to the linear range. Different LC conditions were used for sphingolipid analysis. For GlcCer and galactosylceramide separation, two Waters Atlantis Silica HILIC columns (2.1 × 150 mm, 3 *μ*m particle, Waters Corp., Milford, MA) were used in tandem. For the analysis of LacCer and GM3, Waters Acquity UPLC BEH HILIC column (2.1 × 100 mm 1.7 *μ*m particles) were used. For the analysis of ceramide, a Waters Acquity UPLC BEH C8 column (100 × 2.1 mm 1.7 *μ*m particles) was used. For the analysis of PC and SM, a Phenomenex Luna HILIC column (2.0 × 100 mm 3 *μ*m particles, Phenomenex, Torrance, CA). Ceramide was analyzed with an API‐5000 triple quadrupole tandem mass spectrometer, while the rest were analyzed by an API‐4000 (AB SCIEX, Framingham, MA). All sphingolipids were analyzed in MRM mode except for PC, which was analyzed in a precursor ion mode. Sphingolipid standards were obtained from Matreya, LLC (Pleasant Gap, PA).

### Statistical analysis

Data comparisons were performed using one‐way ANOVA with post analysis using Dunnett's multiple comparison test, two‐way ANOVA with post analysis using Tukey's multiple comparison's test, student's *t*‐test for individual comparisons, or multiple t‐tests using the Holm–Sidak method to correct for multiple comparisons, as indicated; *P* < 0.05 was considered to be a significant difference. Data were expressed as box plots with Tukey‐style whiskers, as the mean ± standard deviation, or mean ± standard error of the mean, as indicated.

## Results

### Timing of *Pkd1* deletion influences the extent and origin of cystogenesis

**Figure 1:**
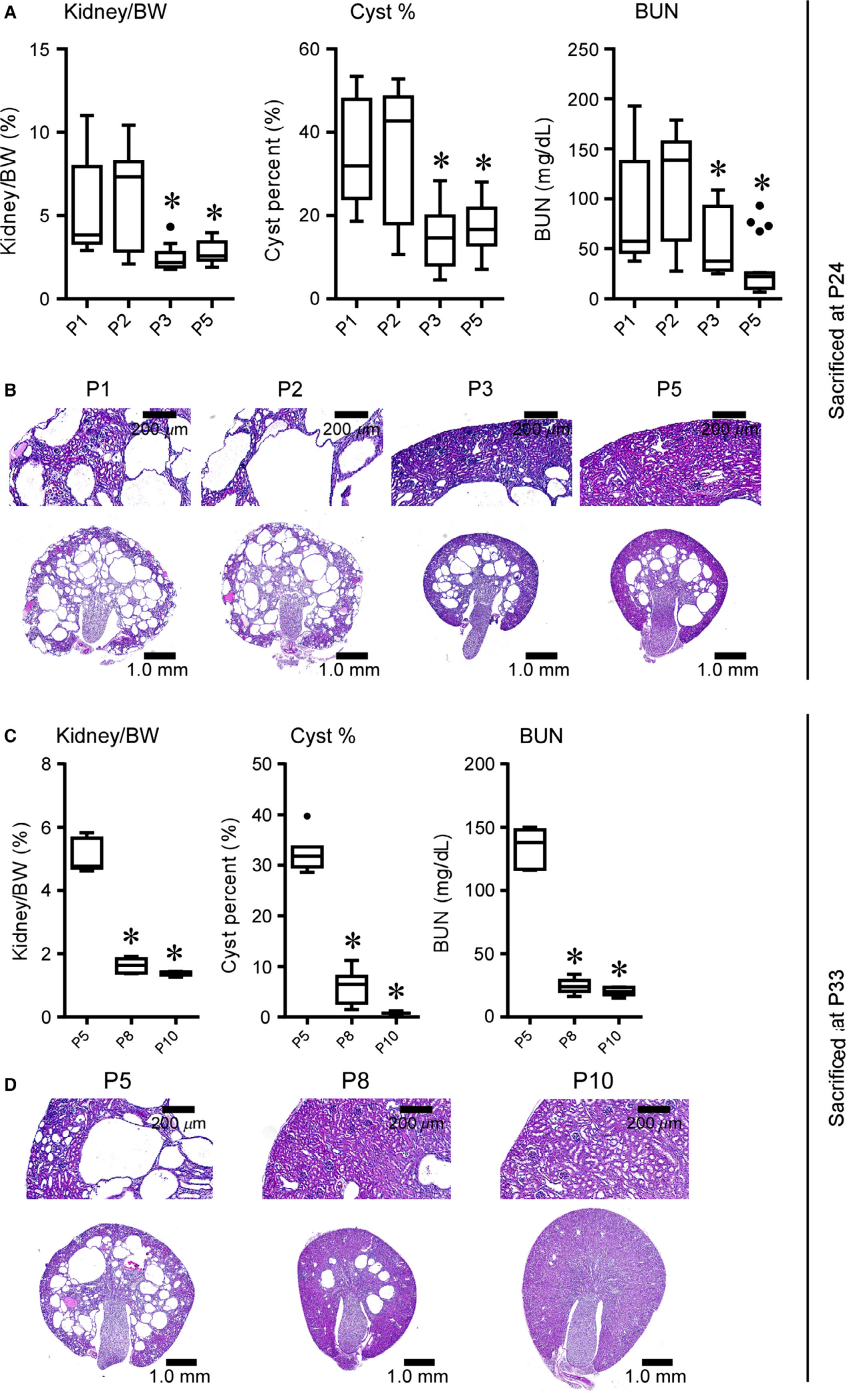
The timing of *Pkd1* deletion modifies the pattern of cyst formation in conditional knockout mice. *Pkd1* cKO mice were exposed to a single 250 mg/kg dose of tamoxifen at the indicated times. Animals were killed at 24 days of age (A–B) or 33 days of age (C–D). (A,C) Quantitative analyses of kidney to body weight ratio (Kidney/BW), cyst percent (Cyst %), and BUN relative to time of conditional *Pkd1* deletion. *P < 0.05 compared to P1 deletion (A) or to P5 deletion (C) using one‐way ANOVA with Dunnett's multiple comparison's test. Data are graphed as box plots with Tukey‐style whiskers. Number of animals, (A): P1, *n* = 31; P2, *n* = 23; P3, *n* = 15; P5, *n* = 21 (B): P5, *n* = 7; P8, *n* = 7; P10, *n* = 9. (B,D) Representative hematoxylin and eosin (H&E)‐stained kidney sections. Note the preservation of cortical tissue and reduced medullary cyst formation in kidneys from P3 or P5 deletions at 24 days as compared to age‐matched kidneys from P1 or P2 deletions (B); progressive reduction in medullary cyst formation is also evident in P8‐ or P10‐deletion kidneys compared to age‐matched P5‐deletion kidneys (D).

We have previously described a neonatal model of ADPKD using these *Pkd1* cKO mice (Natoli et al. 2010). It has been well established that the developmental status of the kidney at the time of *Pkd1* inactivation greatly impacts the resulting cystic phenotype (Lantinga‐van Leeuwen et al. 2007; Piontek et al. 2007). However, these reports have described two phenotypic extremes: deletion before postnatal day 14 yields a rapid, neonatal phenotype, while deletion after day 14 yields a phenotype where cystogenesis is delayed for many months. In an effort to generate a more moderate, adult cystic phenotype that more closely reflects the pathology of ADPKD, we tested the effects of altering the timing and extent of *Pkd1* deletion on the resulting phenotype. To determine how changing the timing of deletion affects cystogenesis, we induced *Pkd1* loss on days 1, 2, 3, 5, 8, and 10. *Pkd1* inactivation on postnatal day 1 (P1) or P2 resulted in significant cystogenesis and loss of kidney function at 24 days of age, as demonstrated by increased kidney/body weight ratio, cyst percent, and BUN (Fig. 1A). Interestingly, although cystogenesis was evident after deletion on P3 or P5, there were significantly fewer cysts compared to deletion on P1 or P2. Furthermore, H&E‐stained kidney sections demonstrated differing patterns of cystogenesis resulting from inactivation on P1 or P2 versus P3 or P5 (Fig. 1B). P1 or P2 deletion resulted in macroscopic and microscopic kidney cyst formation in the cortical and medullary regions of the kidney, whereas only medullary cysts were observed in age‐matched cystic kidneys from P3 or P5 deletion.

To determine if cortical cysts develop later in the course of disease in response to deletion on P5, we extended our analysis to 33 days of age. As expected, kidney/body weight ratio, cyst percent, and BUN increased in severity from 24 to 33 days of age in P5‐deletion mice (compare Fig. 1A and C). Only microcysts and tubular dilations were present in the cortex at 33 days, in contrast to the large cortical cysts obtained after P1 or P2 deletion (Fig. 1B and D). Further delaying *Pkd1* inactivation, with deletion on P8 or P10, yielded progressively milder cystogenesis when compared to P5 deletion, demonstrating a gradual loss of responsiveness in the medulla as well (Fig. 1C–D).

### Tamoxifen dosage influences the severity of cystogenesis

**Figure 2:**
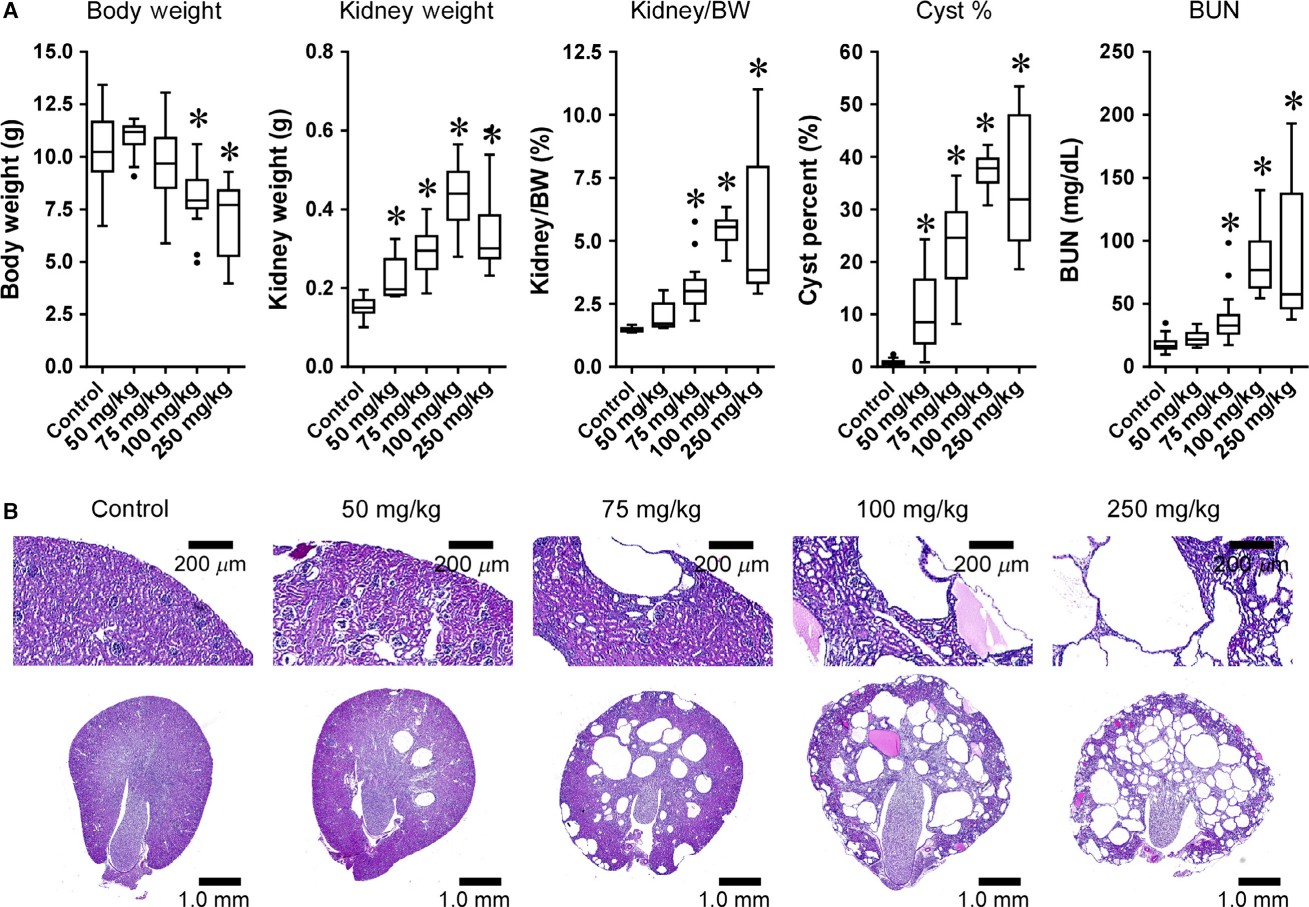
The extent of *Pkd1* inactivation influences the aggressiveness of cystogenesis in conditional knockout mice. The *Pkd1* cKO allele was inactivated on postnatal day 1 using the indicated doses of tamoxifen. Cystogenesis was assessed at 26 days of age. (A) Quantification of body weight, kidney weight, kidney to body weight (Kidney/BW), cyst percentage (Cyst %), and BUN relative to tamoxifen dose. Data are graphed as box plots with Tukey‐style whiskers. *P < 0.05 compared to tamoxifen‐treated littermates lacking the *Pkd1* germline knockout allele (control), using one‐way ANOVA with Dunnett's multiple comparison's analysis. Number of animals: Control, *n* = 38; 50 mg/kg, *n* = 15; 75 mg/kg, *n* = 29; 100 mg/kg, *n* = 14; 250 mg/kg, *n* = 31. (B) Representative hematoxylin and eosin (H&E)‐stained kidney sections. Note the dose‐dependent elevation of cystogenesis generated by increasing the tamoxifen concentration.

In ADPKD, cysts form focally from all nephron segments. The pattern of cortical and medullary cyst formation resulting from P1 or P2 deletion in mice is representative of human cystogenesis, but the rapid rate of cyst formation causes a neonatal phenotype with a rapid loss of kidney function (Fig. 1A). We hypothesized that lowering the dose of tamoxifen on P1 would reduce the number of cysts while still affecting all kidney segments, thus prolonging survival. To test this, tamoxifen was administered on postnatal day 1 at doses of 10, 50, 75, 100, and 250 mg/kg, and pathological assessments were conducted at 26 days of age (Fig. 2A). P1 deletion with 10 mg/kg of tamoxifen did not yield a cystic phenotype (data not shown). Cystogenesis was observed in *Pkd1* cKO mice exposed to 50 mg/kg of tamoxifen, and progressively increased in severity through 100 mg/kg, where it reached a plateau (Fig. 2A). BUN levels at 26 days of age demonstrated that renal function was largely preserved with 50 mg/kg of tamoxifen, and only slightly elevated with a 75 mg/kg dose (Fig. 2A). Kidney histology confirmed the presence of both cortical and medullary cysts at all but the 10 mg/kg tamoxifen dose (Fig. 2B). To determine how altering the tamoxifen dosing regimen influences the extent of deletion, droplet digital PCR was used to quantify the presence of the deleted region of the *Pkd1* gene (between the lox sites) and a region of *Pkd1* outside of the deleted region in kidney DNA samples from treated mice. As shown in Table 1, altering the deletion parameters did influence the extent of deletion. Increasing the concentration of tamoxifen on P1 increased the extent of deletion. Surprisingly, altering the timing of exposure to 250 mg/kg tamoxifen also influenced the extent of deletion. The percentage deletion peaked on P2, then declined; by P8, deletion was achieved in approximately 1% of the cells. P5 deletion percentage was roughly 8.5% in animals killed at either P26 or P33, suggesting that the deletion percentage was stable over this period.

**Table 1:** Percent *Pkd1* deletion in kidneys

Deletion day	Conc. tamoxifen (mg/kg)	Sac age (days)	Ratio inside/outside (mean)	Ratio inside/outside (SD)	% deletion
0	0	64	1.00	0.031	0.00
1	75	26	0.97	0.022	6.15
1	100	26	0.92	0.018	16.33
1	250	26	0.91	0.069	17.79
2	250	26	0.86	0.022	29.11
3	250	26	0.97	0.028	7.53
5	250	26	0.96	0.028	8.73
5	250	33	0.96	0.018	8.52
8	250	33	1.00	0.023	0.82
10	250	33	0.99	0.025	1.74

### Disease progression in *Pkd1* cKO mice deleted on P1 with 75 mg/kg tamoxifen

**Figure 3:**
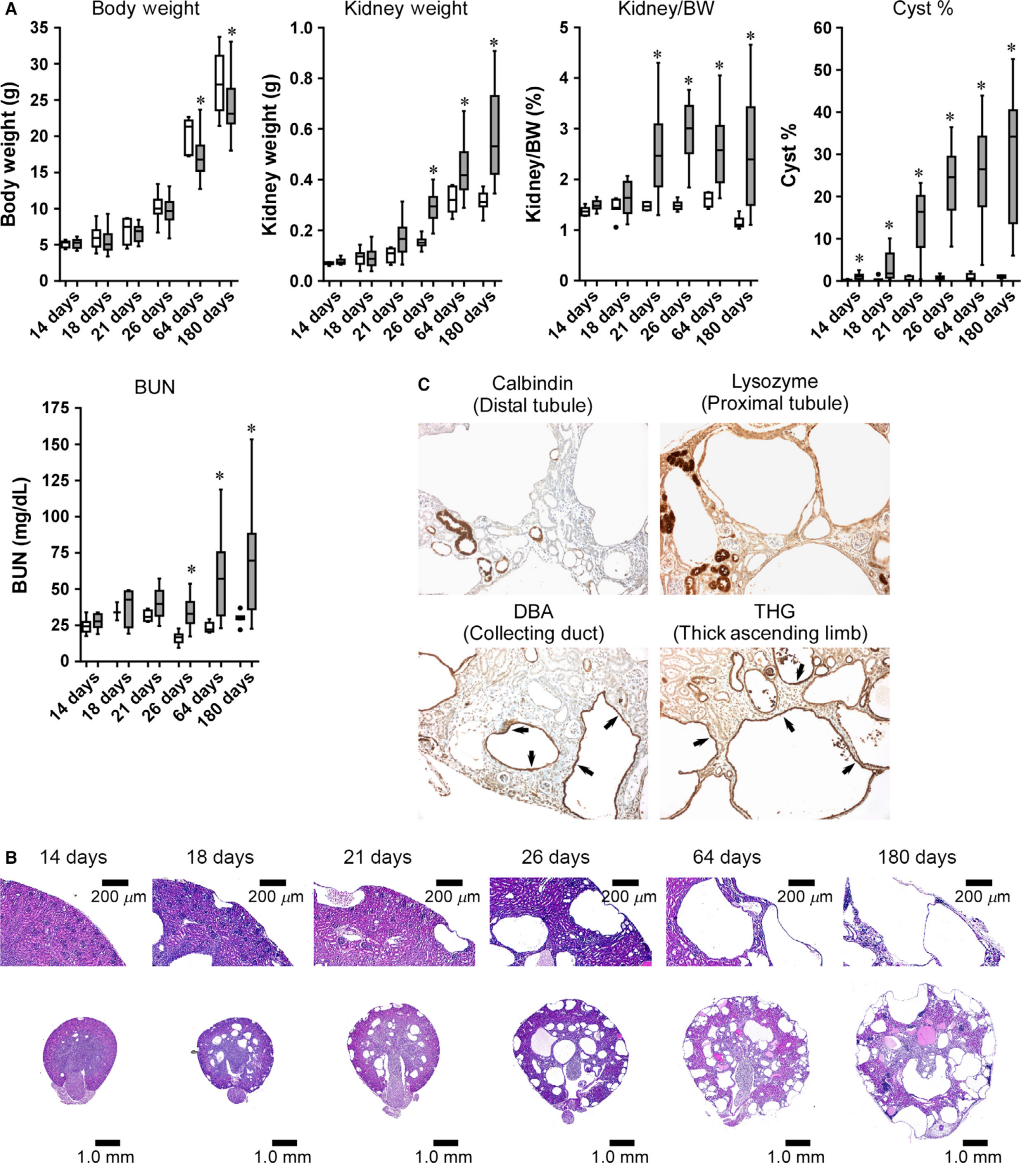
Kinetics of disease progression in a *Pkd1* cKO model of cystogenesis. *Pkd1* cKO mice were exposed to a single 75 mg/kg dose of tamoxifen on postnatal day 1 and killed at the indicated times. (A) Quantitative analyses of body weight, total kidney weight (kidney weight), kidney to body weight ratio (Kidney/BW), cyst percentage (Cyst %), and BUN in cystic *Pkd1* cKO mice (gray boxes) and control littermates (open boxes) relative to age. Data are graphed as box plots with Tukey‐style whiskers. *P < 0.05 compared to age‐matched controls (Student's *t*‐test). Number of animals: Control: 14 days, *n* = 6; 18 days, *n* = 7; 21 days, *n* = 4; 26 days, *n* = 37; 64 days, *n* = 5; 180 days, *n* = 8; cKO: 14 days, *n* = 23; 18 days, *n* = 14; 21 days, *n* = 16; 26 days, *n* = 29; 64 days, *n* = 22; 180 days, *n* = 30. (B) Representative H&E‐stained kidney sections. (C) Immunohistochemical staining of 64‐day‐old cystic *Pkd1* cKO mouse kidney sections with nephron segment‐specific markers following 75 mg/kg tamoxifen exposure on postnatal day 1. Cysts were positively stained for the collecting duct marker Dolichos biflorus agglutinin lectin (DBA) and the thick ascending limb marker Tamm–Horsfall glycoprotein (THG). Cysts were negative for the proximal tubule marker lysozyme and the distal tubule marker calbindin. Arrows point to immunoreactive cystic epithelia.

Since P1 deletion with 75 mg/kg tamoxifen yields a mild cystic phenotype with minimal impairment of renal function by 26 days of age, we used this deletion scheme to further characterize disease progression. Pathological assessments were performed on P1‐deleted, 75 mg/kg tamoxifen‐treated mutant mice and control littermates at 14, 18, 21, 26, 64, and 180 days of age. Body weight gain in tamoxifen‐treated mutant mice is slower than controls, reaching statistical significance by 64 days of age (Fig. 3A). While control kidneys cease growing after 64 days, mutant kidneys continually gain weight through 6 months of age (Fig. 3A). Cyst percentage was slightly but significantly elevated starting at 14 days, and further increased throughout disease progression (Fig. 3A and B). Kidney function was significantly impaired in *Pkd1* cKO mice by 26 days of age (based on a significant BUN elevation in cystic mice vs. controls) and became further exacerbated as disease progressed (Fig. 3A). Analysis of nephron‐specific markers revealed that cysts were positively stained with the lectin *Dolichos biflorus* agglutinin, a collecting duct marker, and Tamm–Horsfall protein, a marker of the thick ascending limb (Fig. 3C). None of the cysts stained with the proximal tubule marker lysozyme or the distal tubule marker calbindin. Comparison of serial sections revealed that some cysts are negative for all markers tested.

To determine the factors contributing to variability within this model, the total variability of data from the 64‐day‐old animals was decomposed into three different components: interlitter variability, intralitter variability, and between‐gender variability. Interlitter differences accounted for the majority of variability (~75–95%, depending on parameter assessed; see supplementary material). Intralitter variability was much smaller (~2–6%). Although between‐gender variability was not evident when considering the populations as a whole, gender contributed nearly 20% of the total variability in cyst percentage, suggesting the possibility of a small but real gender difference in this model that is masked by the interlitter variability.

**Figure 4:**
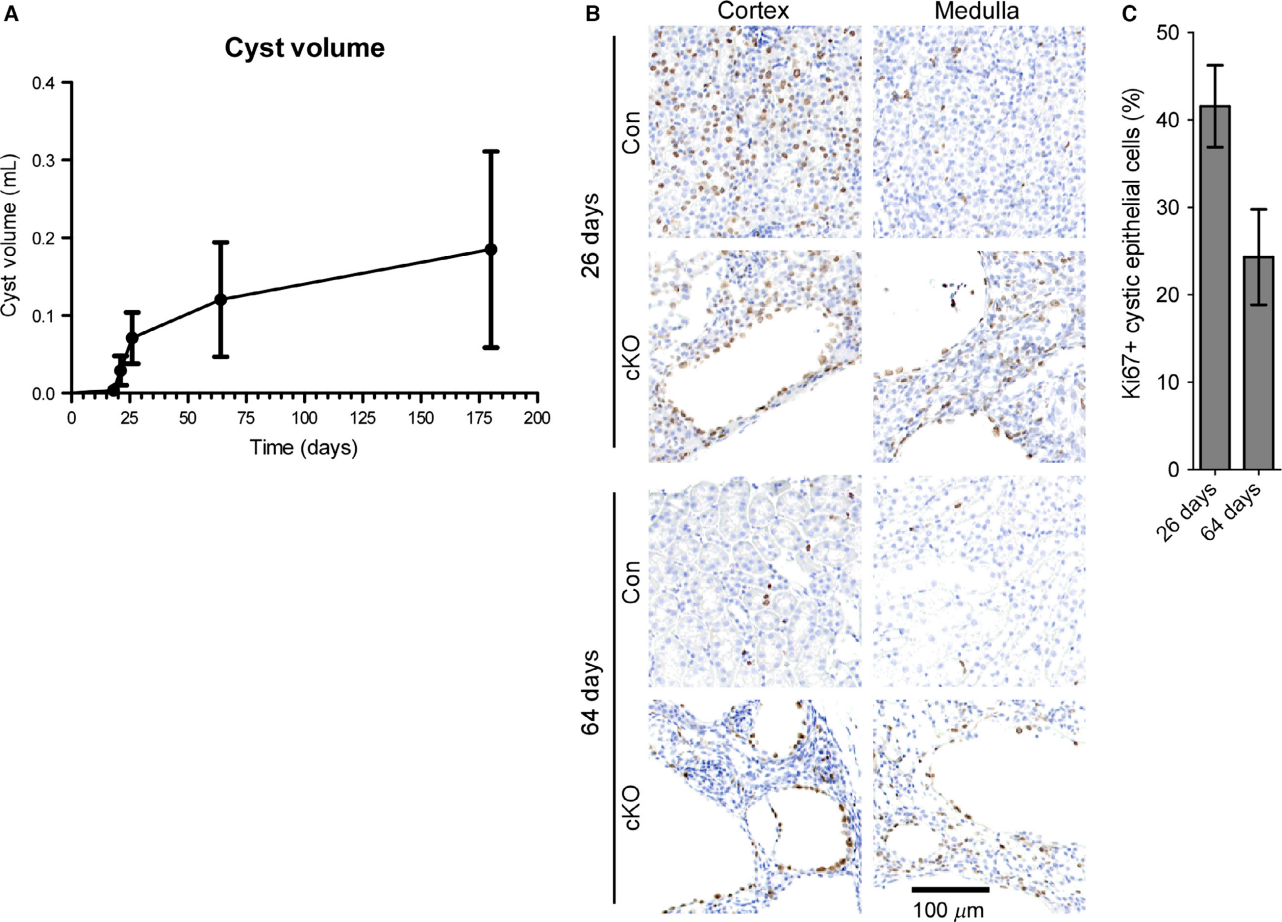
Cyst growth occurs in an early, rapid growth phase followed by a slow growth phase. (A) Cyst volume was estimated at 14, 18, 21, 26, 64, and 180 days of age as described in the materials and methods and plotted as a function of time. The resulting graph shows an early, rapid growth phase from 18 to 26 days of age and a late, slow growth phase from 64 to 180 days of age. (B) Ki67 immunohistochemical staining of kidney sections from *Pkd1* cKO mice at 26 and 64 days of age. Images from both the cortex (left) and medulla (right) are shown. Con, control littermates; cKO,* Pkd1* cKO mice. (C) Quantification of Ki67‐positive cystic epithelial cells at 26 and 64 days of age, expressed as a percentage of the total number of cystic epithelial cells. A total of 40–50 cysts from each of four representative animals per group were quantified. Mean ± SEM are indicated.

The data in Figure 3 suggest that rapid cystogenesis is occurring at the early stages of disease in this model. To more carefully assess the cyst growth rate, we estimated the total cyst volume at each time point as described in the Materials and Methods. As shown in Figure 4A, cyst growth occurs in a biphasic manner; from 18 to 26 days, rapid cyst growth occurs at a rate of approximately 8.4 *μ*L/d, but thereafter, the cyst growth rate slows to approximately 0.6 *μ*L/d. To determine if the difference in cyst growth was due to differences in the rate of cystic epithelial cell proliferation, we performed immunohistochemical staining for the proliferative cell marker, Ki67 (Fig. 4B). A comparison of Ki67‐positive cells in control animals demonstrates a marked increase in Ki67 staining in 26‐day‐old animals compared to 64‐day‐old animals. Quantification reveals a high percentage of Ki67‐positive cyst‐lining epithelial cells at both 26 and 64 days of age (roughly 42% and 25%, respectively; Fig. 4C).

### Dysregulation of molecular pathways in *Pkd1* cKO mice resemble those that occur in human ADPKD

**Figure 5:**
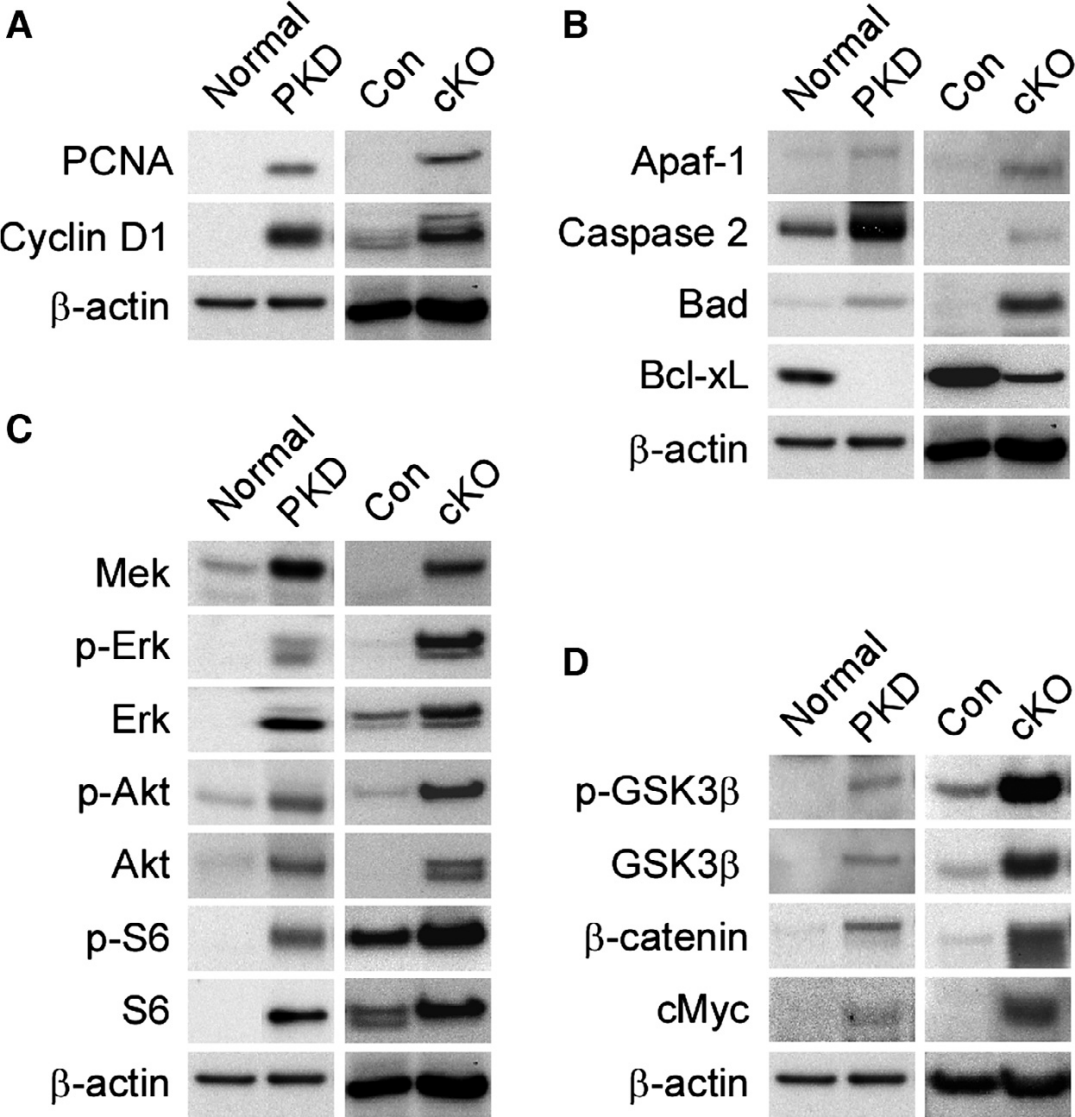
Dysregulation of common molecular pathways drive cystogenesis in human and murine PKD. RIPA extracts were prepared from normal and cystic kidneys from human adults, as well as normal and cystic 64‐day‐old *Pkd1* cKO mice, and assessed for the expression of markers of the cell cycle regulatory, MAPK/ERK, Akt‐mTOR, Wnt/*β*‐catenin or apoptotic pathways. *Pkd1* cKO mice and corresponding littermate controls were exposed to 75 mg/kg tamoxifen on P1. Normal: normal human; PKD: human ADPKD; Con: *Pkd1* control littermate; cKO:* Pkd1* cKO mouse. (A) Western blot analysis of cell cycle markers. (B) Expression levels of apoptosis markers. (C) Analysis of marker expression levels of mitogenic and Akt/mTOR signaling pathways. (D) Status of canonical Wnt signaling pathway.

Cystogenesis in human ADPKD and several mouse models of PKD is associated with dysregulation of multiple biochemical pathways, such as cell‐cycle regulation, apoptosis, cyclic AMP, MAPK/ERK, Akt/mTOR, and canonical Wnt/*β*‐catenin signaling cascades (Takiar and Caplan 2011). To determine if similar mechanisms are associated with cystogenesis in the *Pkd1* cKO model, we compared the expression of markers related to cystogenic pathways in normal or cystic kidneys from humans and *Pkd1* cKO mice. Since elevated kidney proliferation and apoptosis are hallmarks of human ADPKD (Ibrahim 2007), we first assessed the expression of markers involved in these processes (Figs. 4B,C and 5A,B). Cell cycle regulatory proteins and apoptotic markers were similarly dysregulated in kidneys from human ADPKD and *Pkd1* cKO mice (Fig. 5A and B). All cystic samples demonstrated increased expression of the proliferation marker PCNA and the cell cycle regulator cyclin D1 relative to their noncystic controls (Fig. 5A). Likewise, the expression levels of the proapoptotic markers Apaf‐1, Caspase‐2, and Bad were consistently up‐regulated in cystic human and *Pkd1* cKO mouse kidneys, accompanied by down‐regulation of the antiapoptotic marker Bcl‐xL (Fig. 5B). Hyperactive mTOR signaling is a common feature of many forms of PKD (Ibraghimov‐Beskrovnaya and Natoli 2011). We observed a common increase in Akt and S6 phosphorylation in cystic kidneys compared to controls, indicating increased mTOR activity (Fig. 5C). Activation of canonical Wnt/*β*‐catenin signaling has been reported in some model systems of cystic kidney disease (Wuebken and Schmidt‐Ott 2011). Increased levels of GSK3*β* phosphorylation, *β*‐catenin, and cMyc expression in all cystic kidney samples demonstrate that activation of the Wnt/*β*‐catenin signaling pathway is another common feature of human ADPKD and *Pkd1* cKO mice (Fig. 5D).

### Renal fibrosis in *Pkd1* cKO mice

**Figure 6:**
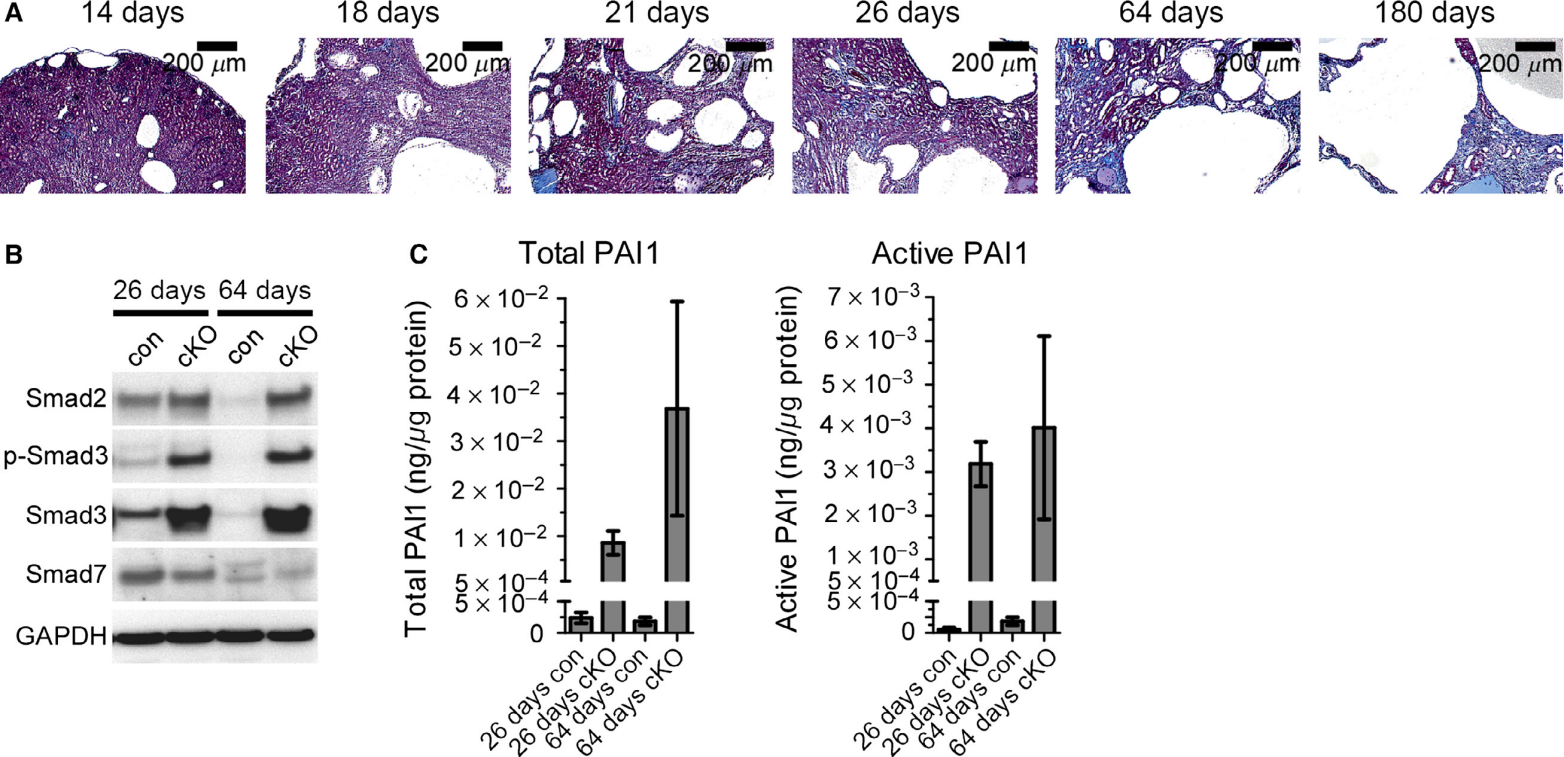
Progression of fibrosis in *Pkd1* cKO mice. *Pkd1* cKO mice and corresponding littermate controls were exposed to 75 kg/kg tamoxifen on P1 and harvested at the indicated time points. (A) Trichrome staining of representative cystic kidneys harvested at 14, 18, 21, 26, 64, or 180 days of age. (B) Western blot analysis of kidney lysates from 26‐ or 64‐day‐old *Pkd1* cKO mice (cKO) or control littermates (con) for TGF‐*β* signaling markers. (C) ELISA analysis of total or active PAI1 from lysates prepared from 26‐ or 64‐day‐old *Pkd1* cKO mouse kidneys (cKO) or control littermates (con).

In ADPKD, up‐regulation of fibrotic signaling pathways causes interstitial kidney fibrosis; interstitial fibrosis in turn influences the onset and rate of renal functional decline to end‐stage renal disease (Norman 2011). We therefore sought to understand the development of fibrosis in the *Pkd1* cKO model. Histological analysis of cystic *Pkd1* cKO kidney sections stained with Masson's trichrome revealed noticeable interstitial fibrosis beginning at 26 days of age, and progressively increased throughout the interstitial area until the majority of the renal parenchyma was fibrotic by 6 months of age (Fig. 6A). Since TGF‐*β* signaling is known to promote fibrosis, we examined the expression and phosphorylation of the profibrotic transcription factors Smad2 and Smad3, and the antifibrotic transcription factor Smad7, as markers of TGF‐*β* activity (Sharma and Ziyadeh 1994; Yamamoto et al. 1994; Shi and Massague 2003). Expression of all Smads was evident at 26 days of age, even in control animals (Fig. 6B). Phosphorylation of Smad3 was also observed at 26 days of age in control animals, suggesting that Smad activation occurs in the neonates in the absence of fibrosis. Cystic animals showed increased expression of Smad2 and Smad3, with decreased expression of Smad7, at both 26 and 64 days of age, confirming TGF‐*β* activation (Fig. 6B). TGF‐*β* signaling can activate PAI1 expression to promote fibrosis (Eddy and Fogo 2006); therefore, we assessed the total and active PAI1 levels in cystic kidneys (Fig. 6C). Both total and active PAI1 levels were extremely low in control kidneys, consistent with a lack of fibrosis. Total and active PAI1 were significantly elevated in *Pkd1* cKO kidneys compared to age‐matched controls. Increased levels of total PAI1 were observed in 64‐day‐old *Pkd1* cKO kidneys compared to 26‐day‐old *Pkd1* cKO kidneys; a similar, albeit smaller, increase was observed for active PAI1. Therefore, kidney fibrosis is underway by 26 days of age in the *Pkd1* cKO model.

### *Pkd1* is required for developmental repression of molecular pathways associated with cystogenesis

**Figure 7:**
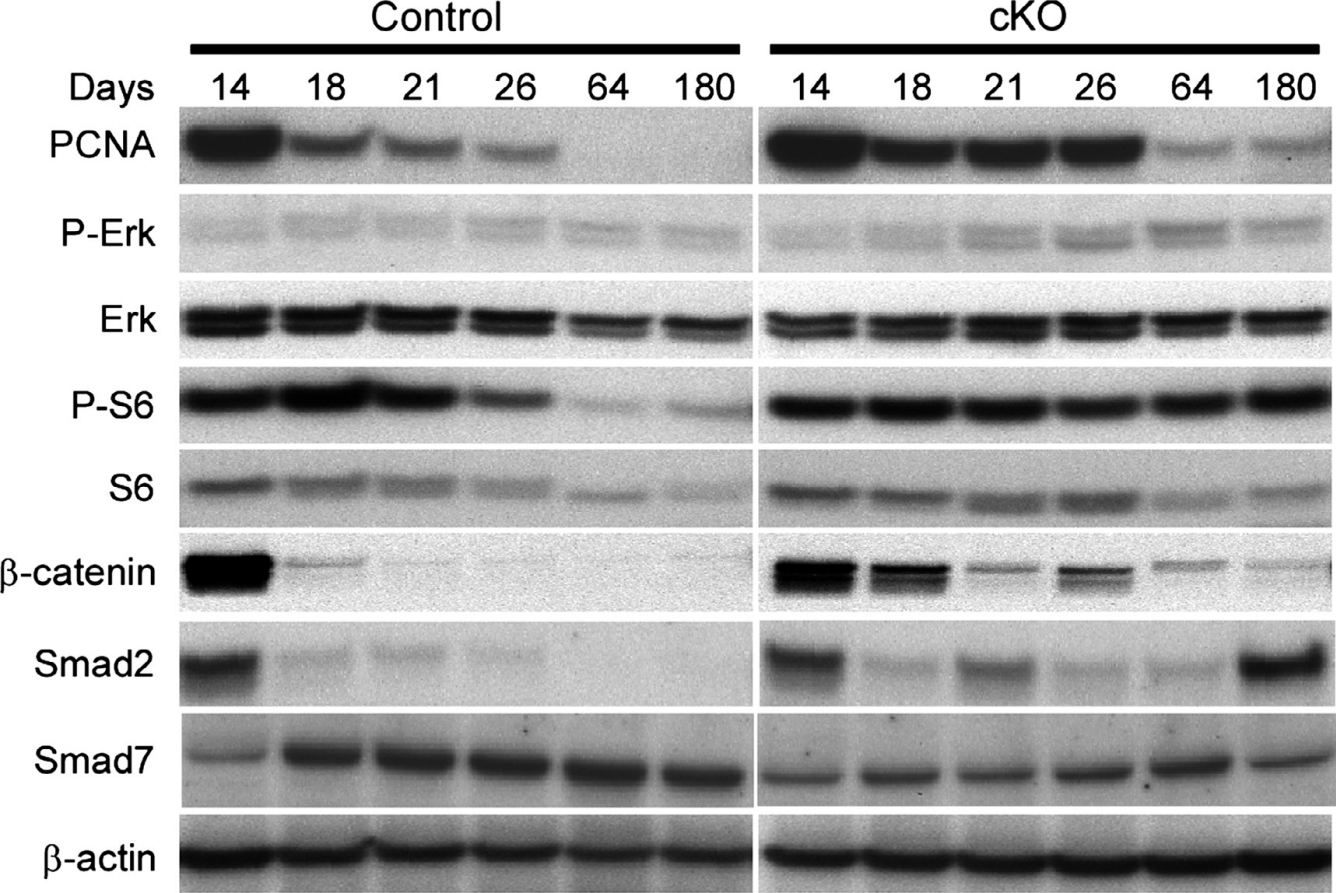
Loss of *Pkd1* sustains expression of markers that are down‐regulated during normal postnatal development. *Pkd1* cKO mice and littermate controls were exposed to 75 mg/kg tamoxifen on postnatal day 1 and the kidneys were harvested at the indicated times for western blot analysis of representative markers of cell cycle regulatory, MAPK/ERK, Akt‐mTOR, and Wnt/*β*‐catenin pathways.

To determine the timing of changes in signaling pathway activity following *Pkd1* deletion, we assessed the behavior of a subset of the dysregulated markers in control and cystic kidneys at 14, 18, 21, 26, 64, and 180 days of age (Fig. 7). The expression of all markers analyzed was equivalent in control and cystic *Pkd1* cKO kidneys at 14 days. PCNA, *β*‐catenin, and Smad2 were elevated in cystic kidneys compared to age‐matched controls by 18 days of age, and the phosphorylated forms of both Erk and ribosomal protein S6 were elevated compared to wild‐type controls by 26 days of age. Although a decrease in PCNA, ribosomal protein S6, and *β*‐catenin levels occurred in cystic kidneys after 26 days, levels of these markers remained elevated compared to age‐matched control kidneys through 180 days of age. A decrease in ribosomal protein S6 phosphorylation did not occur in cystic kidneys, even at 180 days of age, whereas wild‐type kidneys down‐regulate S6 phosphorylation by 26 days of age. An abrupt loss of *β*‐catenin and Smad2 expression was observed between days 14 and 18 in control kidneys, whereas sustained *β*‐catenin and Smad2 expression was observed in the *Pkd1* cKO kidneys. Smad7 is detectable in both control and *Pkd1* cKO kidneys at similar levels at day 14; Smad7 levels rise in control animals at day 18, and the levels remain elevated thereafter. Smad7 levels also rise between days 14 and 18 in *Pkd1* cKO animals, but they do not rise to the same extent as age‐matched controls. The loss of *β*‐catenin expression in the control animals correlates with the onset of rapid cystogenesis in the cystic animals. These data suggest that cell cycle regulatory, mitogenic signaling, mTOR signaling, Wnt/*β*‐catenin, and TGF‐*β* signaling pathways are active during neonatal mouse development, and that *Pkd1* function is required to suppress their activation.

### Increased kidney glycosphingolipid accumulation initiates at the onset of cystogenesis in *Pkd1* cKO mice

**Figure 8:**
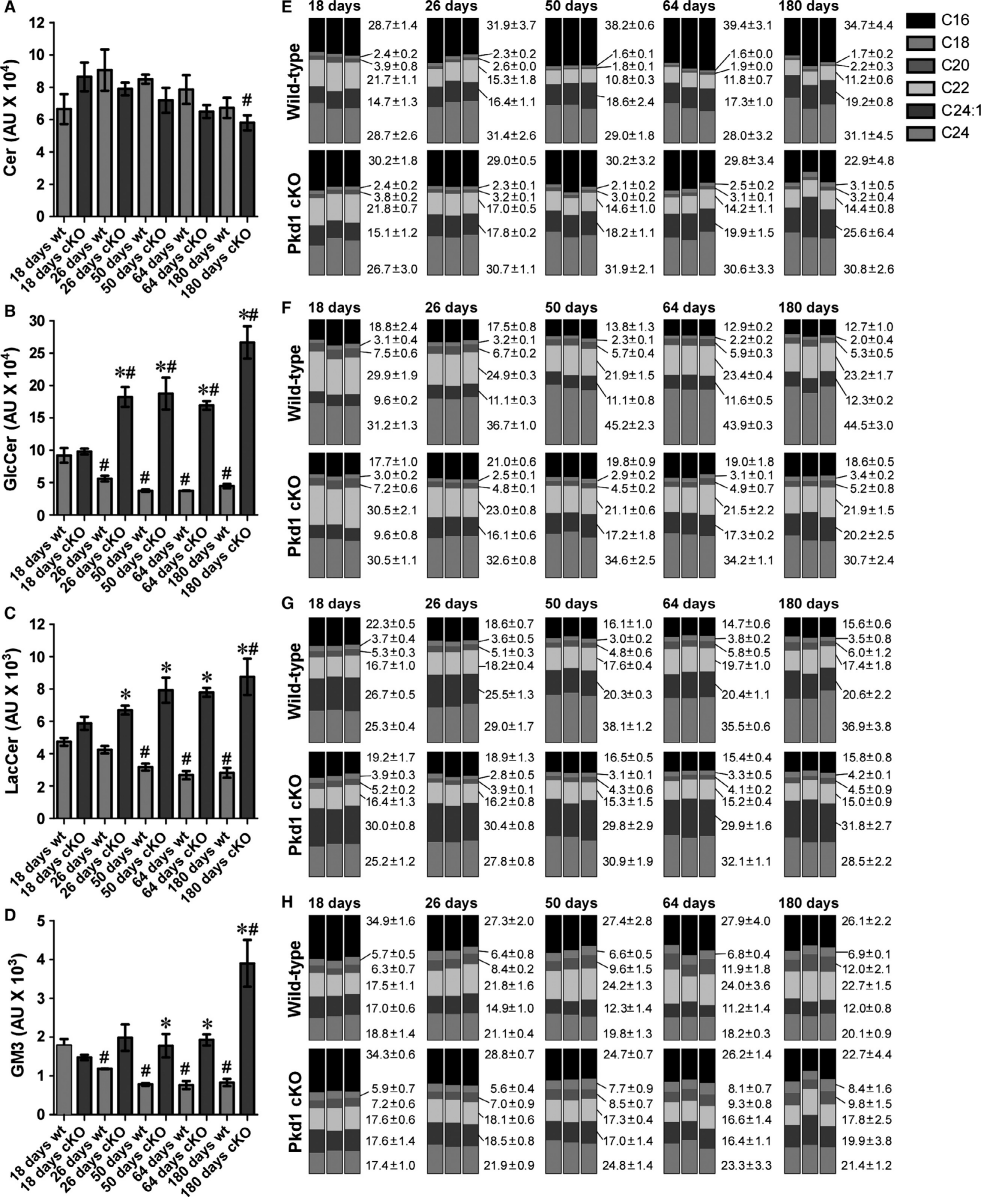
Dysregulated glycosphingolipid metabolism is evident shortly after the onset of cystogenesis. Kidney lysates from three individual control or *Pkd1* cKO mice treated with 75 mg/kg tamoxifen on postnatal day 1 were analyzed by LC/MS/MS for ceramide (Cer), glucosylceramide (GlcCer), lactosylceramide (LacCer), and GM3 content; in all cases, the C16, C18, C20, C22, C24:1, and C24 isoforms were measured. (A–D) Relative abundance of total ceramide (A), GlcCer (B), LacCer (C), and GM3 (D) is shown for 18‐, 26‐, 50‐, 64‐, and 180‐day‐old *Pkd1* cKO (cKO, dark gray boxes) or control littermates (wt, light gray boxes) as indicated. Data are presented as mean ± SEM. Changes in glycosphingolipid levels over time for a given genotype were assessed using one‐way ANOVA with a Dunnett's *t*‐test for multiple comparisons adjustment. Comparisons between age‐matched *Pkd1* cKO animals and control animals were performed using a Student's *t*‐test. *P < 0.05 compared to age‐matched controls. ^#^ *P* < 0.05 compared to P18 genotype‐matched group. (E–H) Relative percentage of individual acyl chain length isoforms for ceramide (E), glucosylceramide (F), lactosylceramide (G), and GM3 (H) are shown as stacked bar graphs. Each stacked bar graph presents the results from an individual animal; mean ± SD for the control (wild‐type) and *Pkd1* cKO mice (Pkd1 cKO) for each time point are indicated to the right of the each set. Since it is difficult to perform statistical analysis of constrained data, no statistical analysis of this data was performed.

Elevated kidney levels of the glycosphingolipids glucosylceramide (GlcCer), lactosylceramide (LacCer), and ganglioside GM3 (GM3) have been reported in human ADPKD and a number of murine models of PKD (Deshmukh et al. 1994; Chatterjee et al. 1996; Natoli et al. 2010). To determine the timing of glycosphingolipid metabolic dysregulation following *Pkd1* deletion, kidney ceramide (the precursor to these glycosphingolipids), GlcCer, LacCer, and GM3 levels were analyzed by LC/MS/MS in control and *Pkd1* cKO mice treated with 75 mg/kg tamoxifen on P1 (Fig. 8). Kidney ceramide levels do not significantly change with age for either the control or *Pkd1* cKO animals (Fig. 8A). Additionally, the only significant difference in kidney ceramide levels in *Pkd1* cKO animals compared to age‐matched controls occurred in 180‐day‐old animals. At 18 days of age, there is no significant difference in the kidney glycosphingolipid levels between *Pkd1* cKO animals and controls (Fig. 8B–D). In control kidneys, glycosphingolipid levels decrease between 18 and 26 days of age, and reach a stable level by 50 days of age (Fig. 8B–D, light gray bars). In contrast, glycosphingolipid levels in *Pkd1* cKO kidneys increase from 18 to 26 days, and remain elevated thereafter (Fig. 8B–D, dark gray bars). As a result, by 26 days of age, there is a significant increase in the levels of GlcCer and LacCer in *Pkd1* cKO animals compared to their age‐matched controls, and by 50 days of age, all glycosphingolipids are elevated in *Pkd1* cKO animals compared to their age‐matched controls. Therefore, dysregulation of glycosphingolipid metabolism is evident shortly after the onset of cystogenesis.

Although increased glycosphingolipid levels have been well documented in PKD, there have been no reports assessing changes in composition of the acyl chains that comprise the ceramide backbone of these glycosphingolipids. Acyl chain length is an important factor regulating the interaction of sphingolipids with proteins or other lipids, and distinct acyl chains can be generated independently through the action of unique ceramide synthases (Malinina et al. 2006; Iwabuchi et al. 2010; Mullen et al. 2011; Grosch et al. 2012). We therefore determined the relative contribution of the individual chain length isoforms for each lipid (measured as a percentage of the total, to control for differences in total abundance). As shown in Figure 8B, the changes in isoform composition are much more subtle than the changes in total abundance, and suggest both age‐ and genotype‐dependent differences; however, due to the constrained nature of the data, it is difficult to determine the statistical significance of the results.

**Figure 9:**
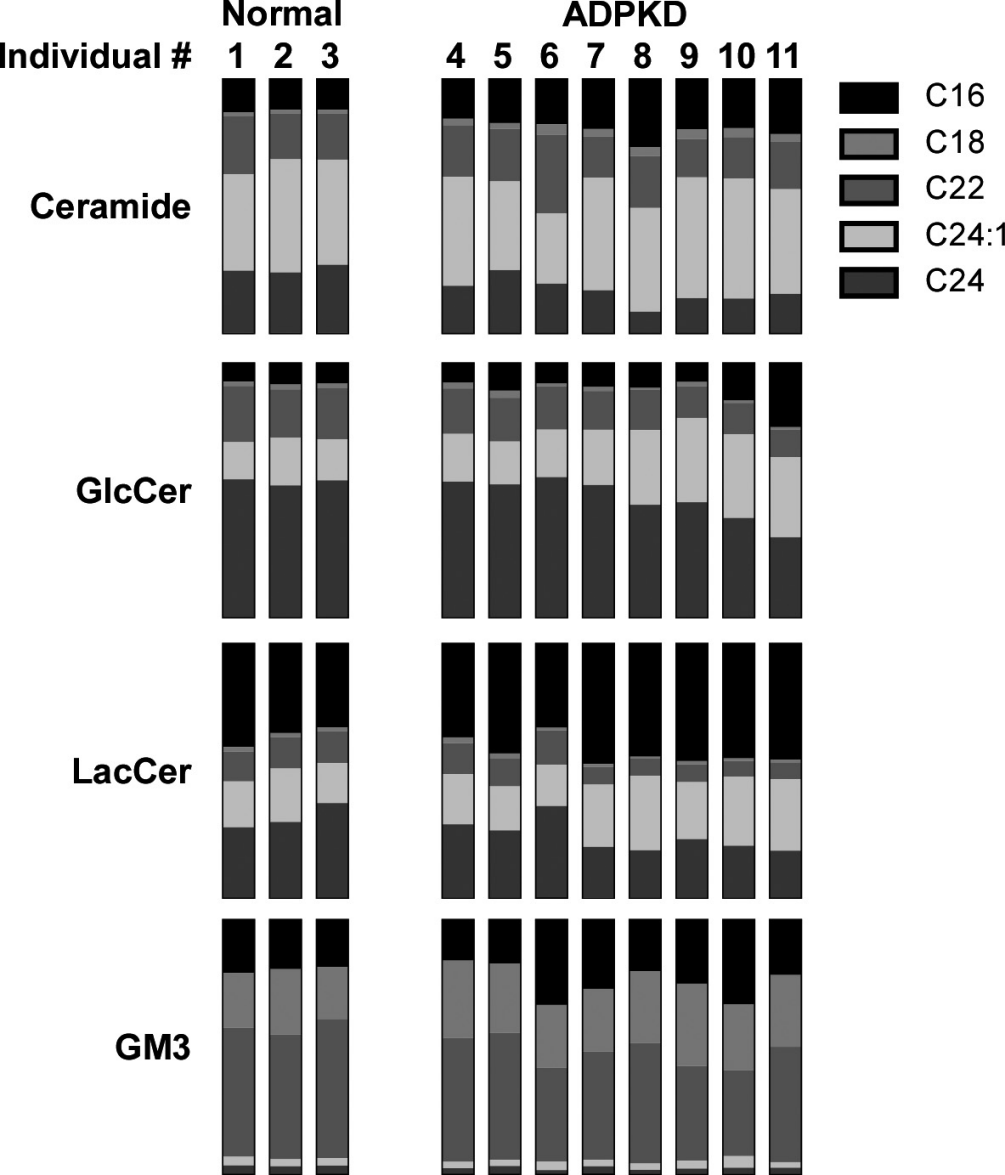
Glycosphingolipid acyl chain lengths are minimally altered in human ADPKD. Kidney lysates from three normal individuals and eight individuals with ADPKD were analyzed by LC/MS/MS for ceramide (Cer), glucosylceramide (GlcCer), lactosylceramide (LacCer), and GM3 isoform content. Stacked bar graphs showing the relative percentage of distinct acyl chain length isoforms for ceramide (E), glucosylceramide (F), lactosylceramide (G), and GM3 (H) are presented for each individual. Results from a single individual are organized in a single column. The C20 isoform could not be measured in these samples due to low abundance.

We and others have reported increased kidney GlcCer, LacCer, and GM3 in human ADPKD, but there have been no reports on analysis of changes in isoform composition in ADPKD patients. To address this, we have assessed the isoform composition in human ADPKD samples (Fig. 9). We have previously reported an increase in total glycosphingolipid levels in these samples (Natoli et al. 2010). In human cystic kidneys, the isoform composition either resembles the isoform composition present in normal kidneys, or subtly trends toward the isoform changes seen in the *Pkd1* cKO mice. This demonstrates that the changes in glycosphingolipid metabolism occurring in human cystic kidneys are accurately reflected in the mouse *Pkd1* cKO model.

### Liver disease manifestations in *Pkd1* cKO mice

**Figure 10:**
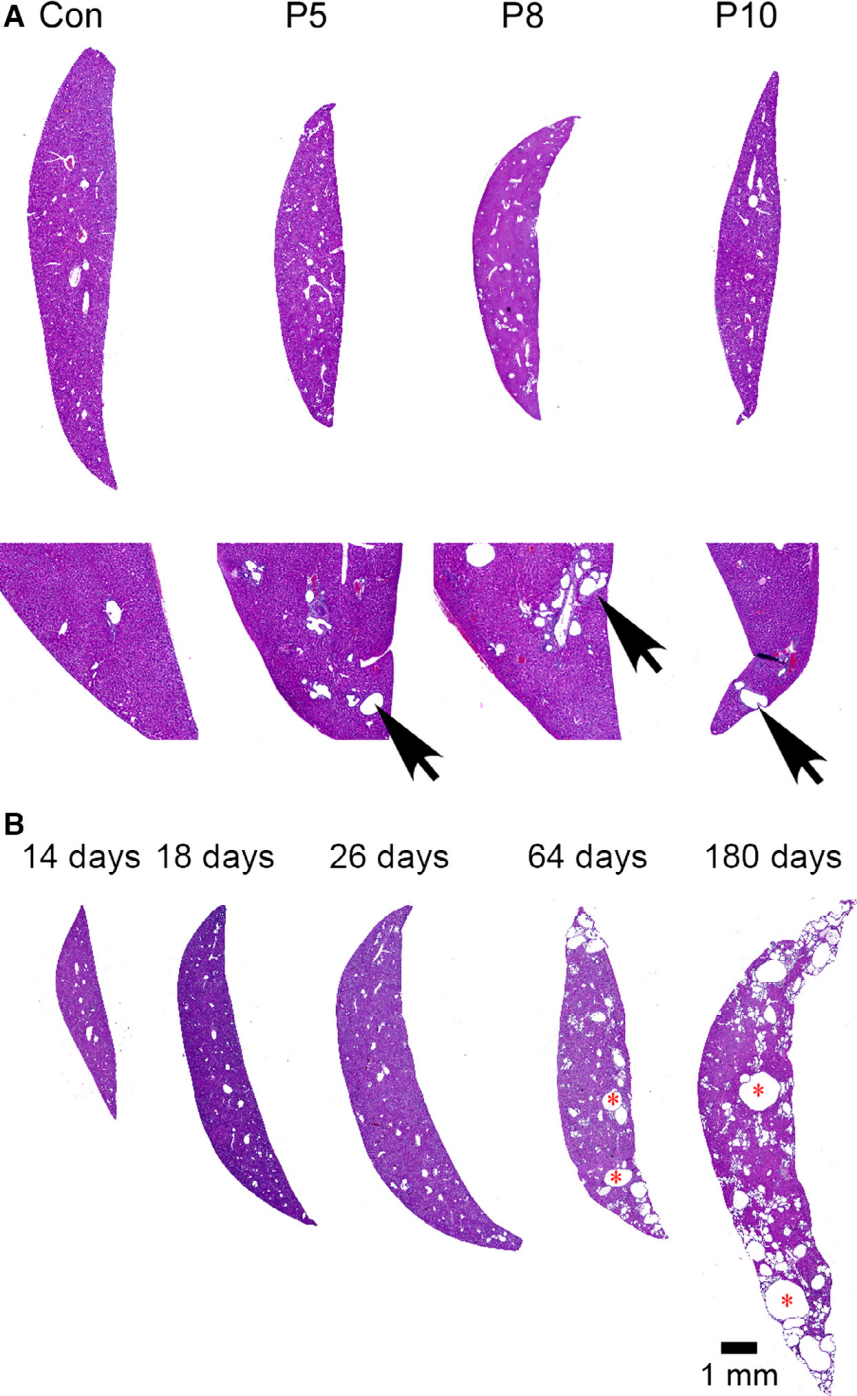
Development of liver cysts in a *Pkd1* cKO mouse model. (A) Control (Con) or *Pkd1* cKO mice were exposed to 250 mg/kg tamoxifen on postnatal day 5, 8, or 10 as indicated above in the photographs. Animals were killed at 33 days of age for analysis of liver histology. The lower images are high‐resolution versions of the image above. Arrows indicate cystic regions. (B) *Pkd1* cKO mice were exposed to 75 mg/kg tamoxifen on postnatal day 1 and killed at the indicated times for analysis of liver histology. Microscopic liver cysts are evident at postnatal day 14, and progressively enlarge throughout 6 months of age. *liver cyst lumen.

Since 85% of ADPKD patients between 25 and 34 years of age develop liver cysts (Pirson 2010), we assessed liver cyst formation in *Pkd1* cKO mice (Fig. 10). To determine if liver cyst formation is sensitive to the timing of *Pkd1* loss, we analyzed cyst formation following *Pkd1* deletion using 250 mg/kg tamoxifen on P5, P8, and P10 (Fig. 10A). Later deletion did not noticeably influence liver cyst formation or growth, confirming prior reports (Lantinga‐van Leeuwen et al. 2007; Piontek et al. 2007). We next examined the progression of liver cystogenesis following *Pkd1* deletion on P1 using 75 mg/kg tamoxifen (Fig. 10B). Cystic expansion of biliary ducts is microscopically observable beginning at 14 days of age (not shown) and grossly evident by 64 days. However, levels of the enzymes alanine aminotransferase and aspartate aminotransferase were not abnormally elevated, even at late time points, indicating that liver function is not significantly impacted, despite extensive cystogenesis (at 6 months, aspartate aminotransferase = 239.4 ± 78 U/L for control mice, 213.5 ± 25.9 U/L for cystic mice, normal range = 72–288 U/L; alanine aminotransferase = 56.9 ± 6.9 U/L for control mice, 56.1 ± 3.1 U/L for cystic mice, normal range = 24–140 U/L). Although pancreatic cysts are reported in about 9% of ADPKD patients, we have not observed pancreatic cysts in *Pkd1* cKO animals with any of the deletion schemes used (Pirson 2010).

## Discussion

Here, we report the generation of a *Pkd1*‐linked, inducible mouse model that represents the genetic and phenotypic characteristics of human ADPKD, and demonstrate that subtle differences in the timing and extent of *Pkd1* loss cause significant changes in the pattern of kidney cyst growth. The pathological similarities between *Pkd1* cKO disease and ADPKD include renal manifestations such as progressive cyst growth accompanied by fibrosis and loss of kidney function, as well as liver cyst formation. Comparison of the molecular changes occurring in cystic kidneys reveals a common set of dysregulated pathways in human and murine PKD. Furthermore, analysis of the timing of the changes in pathway activity suggests that loss of polycystin function interferes with the normal developmental regulation of these key pathways.

Neonatal loss of *Pkd1* in the first 2 weeks of life is known to produce rapid and aggressive cyst growth within the developing mouse kidney (Lantinga‐van Leeuwen et al. 2007; Piontek et al. 2007; Takakura et al. 2008). Neonatal inactivation of *Pkd1* within the first 3 weeks of life identified a developmental switch for aggressive cyst growth by 12 days of age; a similar developmental switch has been demonstrated in the *Hnf1b* and *Kif3a* conditional knockout mouse models of kidney cystogenesis, suggesting a common pathway for cystogenesis (Piontek et al. 2007; Patel et al. 2008; Verdeguer et al. 2010). Our results demonstrate that differences in cortical and medullary cyst formation result from subtle changes in the timing of *Pkd1* deletion. Loss of *Pkd1* on P1 or P2 yields extensive cortical and medullary cyst formation, while *Pkd1* inactivation on P3 or P5 manifests as a less aggressive disease resulting mainly from medullary cysts. Although rare cortical cysts can be observed in response to P5 deletion, they are far smaller than the corresponding cortical cysts resulting from deletion on P1 or P2, even when given more time to develop. This suggests that the developmental switch does not occur uniformly throughout the kidney, but rather that different populations of cells undergo the developmental switch at different times. Kidney development and maturation is a complex and heterogeneous process which, in the mouse, continues into the neonatal period (Little and McMahon 2012). Therefore, it is not surprising that subtle changes in the timing of *Pkd1* deletion would elicit differing responses in distinct cell populations.

We have also examined the effect of altering the extent of *Pkd1* deletion on cyst growth by manipulating the dose of tamoxifen. In ADPKD, renal cysts initially form in a focal manner from a small fraction of the total nephrons and gradually increase in size and number (Grantham et al. 1987). The focal nature of cystogenesis has been explained by a “two‐hit” mechanism, involving cyst formation from a subset of nephrons that are affected by spontaneous somatic mutations within the wild‐type *PKD1* or *PKD2* genes on a background of an existing germline mutation (Qian et al. 1996; Qian and Germino 1997). Conditional alleles of *Pkd1* and *Pkd2* in mice can be used to mimic the somatic hits that trigger cyst formation in humans (Wu et al. 1998; Jiang et al. 2006; Lantinga‐van Leeuwen et al. 2007; Piontek et al. 2007; Takakura et al. 2008). Here, we demonstrate that increasing the dose of tamoxifen increases the severity of cyst formation, suggesting a direct relationship between *Pkd1* loss and the extent of cystogenesis. This strategy may be useful for modulating the severity of other diseases that involve loss of heterozygosity, such as cancer.

One limitation of inducible systems is that the chemicals used to activate conditional gene expression may modify disease progression in their own right. For example, doxycycline exacerbates kidney cyst growth in the CD1^*pcy/pcy*^ mouse, but reduces cyst growth in the PCK rat model of PKD (Osten et al. 2009; Liu et al. 2012). The influence of tamoxifen on renal cyst growth has not been explored, but chronic tamoxifen administration has been reported to either positively or negatively impact kidney health. Rats fed a diet containing 250–500 ppm tamoxifen for 18 months develop carcinogen‐related DNA adducts in the kidney that could promote renal damage and cyst growth (Li et al. 1997). In contrast, daily administration of 10 mg/kg tamoxifen has an antifibrotic effect in the hypertensive NAME rat, and reduces glomerulosclerosis (Delle et al. 2012). Since we are delivering a single dose of tamoxifen to nursing females, this should not provide a chronic source of tamoxifen based on the known pharmacokinetics of tamoxifen (and its bioactive derivatives 4‐hydroxytamoxifen and endoxifen) in the mouse (Reid et al. 2014; Binkhorst et al. 2015). The plasma half‐life of tamoxifen and endoxifen in adult mice is 6–7 h after a single oral dose of 20–200 mg/kg tamoxifen; a 200 mg/kg dose is completely cleared of all bioactive forms within 2–3 days (Robinson et al. 1991). While we have not directly assessed kidney tamoxifen exposure, our dosing regimen should have allowed tamoxifen clearance prior to the onset of cystogenesis assuming that metabolism and clearance of tamoxifen is comparable in the neonatal and adult mice. However, it will be necessary to treat mice carrying hypomorphic mutations in the *Pkd1* gene with tamoxifen to definitively determine if tamoxifen alone can alter disease progression.

The concept of the developmental switch is based on data suggesting that the deletion efficiency is not affected by altering the timing of deletion. In our hands, the timing of deletion did influence the extent of *Pkd1* deletion. A similar phenomenon has been reported previously (Lantinga‐van Leeuwen et al. 2007). Other groups have reported that the timing of deletion does not influence the extent of deletion; we note that in these cases, the deletion efficiency was determined using reporters of Cre recombinase activity inserted into the *Rosa26* locus rather than the *Pkd1* gene itself (Piontek et al. 2007; Patel et al. 2008; Verdeguer et al. 2010). Therefore, changes in the severity and pattern of cyst formation could reflect differences in the expression or activity of Cre recombinase, changes in the chromatin organization of the *Pkd1* gene modifying its susceptibility to Cre recombinase activity, or some combination of the two.

The results presented here demonstrate a lag period of up to 17 days between *Pkd1* loss and initiation of cystogenesis, even if *Pkd1* is deleted within the period preceding the developmental switch. When *Pkd1* is deleted on P1, cyst growth does not initiate until day 14–18. The onset of cystogenesis coincides with a period where a number of biochemical changes are occurring in wild‐type mice. At postnatal day 18, a rapid decline of PCNA, *β*‐catenin, and Smad2 is evident, while Smad7 expression increases, suggesting that changes in cell cycle regulation, Wnt signaling, and TGF‐*β* signaling are occurring. Around 26 days of age, ribosomal protein S6 phosphorylation decreases, demonstrating a reduction in mTOR signaling at this point. These changes are delayed in cystic animals, and these pathways are thought to drive cystogenesis (Nadasdy et al. 1995; Yamaguchi 2004; Bukanov et al. 2006; Omori et al. 2006; Shillingford et al. 2006; Ibrahim 2007). These data support the “maturation arrest” hypothesis of cystogenesis, which speculates that *Pkd1* is required for kidney epithelial cell differentiation (Calvet 1994; Piontek et al. 2007).

Also consistent with a maturation arrest hypothesis are the changes in glycosphingolipid composition, with the chain length isoforms in cystic kidneys resembling those observed in juvenile kidneys. However, the observation that glycosphingolipid levels actually increase in cystic kidneys suggests that maturation arrest does not provide a complete picture. If maturation arrest were the only phenomenon occurring in the cystic kidneys, the simple prediction would be that glycosphingolipid levels remain constant, instead of increasing. The onset of the rise in glycosphingolipid levels corresponds to a period of rapid cyst growth between 18 and 26 days of age; this supports a link between dysregulated glycosphingolipid metabolism and cyst growth (Deshmukh et al. 1994; Chatterjee et al. 1996; Natoli et al. 2010). Additionally, it supports the contention that increased glycosphingolipid levels are present in the kidney epithelial cells, rather than being a consequence of an inflammatory response to kidney damage or fibrosis (Chatterjee et al. 1996; Natoli et al. 2010).

When estimated cyst volume is plotted against time, an early period of rapid cyst growth is evident from 18 to 26 days of age, followed by a subsequent period of slow cyst growth. Recent MRI analysis of human cysts, combined with modeling of growth rate estimates, has suggested that there should be a period of extraordinary cyst growth during embryogenesis to account for the observable childhood cyst sizes (Grantham et al. 2010). This rate is thought to significantly exceed the observable cyst growth rates in adults. Kidney development in humans is largely complete by 34 weeks of gestation, but proceeds for 2–3 weeks postpartum in the mouse (Haycock 1998; Grantham et al. 2010); therefore, the period of rapid growth we observe may correspond to the period of rapid growth hypothesized during embryogenesis in human ADPKD. Alternatively, it is possible that small cysts (below the 2 mm limit of detection by MRI) grow at a rapid pace regardless of whether they are formed in utero or in adults, and that the growth rate slows as the cysts enlarge. It is unclear if similar mechanisms regulate the rate of cyst growth in these two stages. In the *Pkd1* cKO mice, the period of rapid growth (18–26 days) is accompanied by a high rate of proliferation, as indicated by PCNA and Ki67 expression. The slower growth period (>26 days) is marked by a lower level of proliferation when compared to the rapid growth period, although it is still elevated compared to normal animals. It is unclear if the differences in proliferation are evident at these two stages of cyst growth are contributing to the different growth rates, or if they simply reflect the differences in proliferation rates evident in age‐matched wild‐type kidneys. Ribosomal protein S6 phosphorylation appears similar at both stages, suggesting that differences in mTOR activity do not account for the difference in growth rate. A better understanding of this issue is necessary to determine the best way to test potential therapies in this model. Despite these concerns, the pathological and biochemical similarities between human ADPKD and this mouse model of PKD suggest that this model could be useful for therapeutic testing.

## Conflict of Interest

The authors were all employees of Sanofi‐Genzyme during the course of this work.

## Supporting information




**Data S1.** Quantification of sources of variability.
