# Supplementary material for: Differences in the timing and magnitude of Pkd1 gene deletion determine the severity of polycystic kidney disease in an orthologous mouse model of ADPKD
Source: Physiol Rep. 2016 Jun 29;4(12):e12846. doi: 10.14814/phy2.12846 (PMC4926022; doi:10.14814/phy2.12846)
Supplement: Supplementary file 1 — Data S1. Quantification of sources of variability. [file PHY2-4-e12846-s001.docx]

A random-effect model is used to quantify the inter- and intra-litter variability. Gender and litter are included in the model. Gender is coded as 1 for female and 2 for male.

$$y_{ijk}=\mu+\alpha_{i}+\beta_{j}+e_{ijk}$$

Where $y_{ijk}$ is the value of the dependent variable for the k-th animal in the j-th litter with gender i, $\mu$ is grand mean, $\alpha_{i}\sim N\left( 0, \sigma_{\alpha}^{2} \right)$ is the random effect for gender $i=1,2$, $\beta_{j}\sim N\left( 0, \sigma_{\beta}^{2} \right)$ is the random effect for litter $j=1,2,\ldots7$, and $e_{ijk}\sim N\left( 0, \sigma^{2} \right)$ represents the intra-litter effect $k=1,2,\ldots, n_{j}$, $n_{j}$ the sample size for the j-th litter. The intra-litter effect includes within-litter between-animal variability, measurement error and other unexplained noises.

The total variability is ${\sigma_{\alpha}^{2}+\sigma_{\beta}^{2}+ \sigma}^{2}$. The % variability for gender, inter-litter and intra-litter is calculated as $100{*\sigma_{\alpha}^{2}}/{{{(\sigma}_{\alpha}^{2}+\sigma_{\beta}^{2}+\sigma}^{2}})\%$, $100{*\sigma_{\beta}^{2}}/{{{(\sigma}_{\alpha}^{2}+\sigma_{\beta}^{2}+\sigma}^{2}})\%$, and $100{*\sigma^{2}}/{{{(\sigma}_{\alpha}^{2}+\sigma_{\beta}^{2}+\sigma}^{2}})\%$ respectively.

## Results:

### BUN:

| Source | Variability % |
| --- | --- |
| Inter-litter | 96.790602 |
| Gender | 1.067356 |
| Intra-litter | 2.142042 |

### Cyst%:

| Source | Variability % |
| --- | --- |
| Inter-litter | 76.562440 |
| Gender | 19.303939 |
| Intra-litter | 4.133621 |

### Ratio K/BW:

| Source | Variability % |
| --- | --- |
| Inter-litter | 92.111532 |
| Gender | 1.279771 |
| Intra-litter | 6.608697 |
